# A Hybrid Least Squares and Principal Component Analysis Algorithm for Raman Spectroscopy

**DOI:** 10.1371/journal.pone.0038850

**Published:** 2012-06-18

**Authors:** Dominique Van de Sompel, Ellis Garai, Cristina Zavaleta, Sanjiv Sam Gambhir

**Affiliations:** Molecular Imaging Program at Stanford (MIPS), Stanford University School of Medicine, Stanford University, Stanford, California, United States of America; University of Quebect at Trois-Rivieres, Canada

## Abstract

Raman spectroscopy is a powerful technique for detecting and quantifying analytes in chemical mixtures. A critical part of Raman spectroscopy is the use of a computer algorithm to analyze the measured Raman spectra. The most commonly used algorithm is the classical least squares method, which is popular due to its speed and ease of implementation. However, it is sensitive to inaccuracies or variations in the reference spectra of the analytes (compounds of interest) and the background. Many algorithms, primarily multivariate calibration methods, have been proposed that increase robustness to such variations. In this study, we propose a novel method that improves robustness even further by explicitly modeling variations in both the background and analyte signals. More specifically, it extends the classical least squares model by allowing the declared reference spectra to vary in accordance with the principal components obtained from training sets of spectra measured in prior characterization experiments. The amount of variation allowed is constrained by the eigenvalues of this principal component analysis. We compare the novel algorithm to the least squares method with a low-order polynomial residual model, as well as a state-of-the-art hybrid linear analysis method. The latter is a multivariate calibration method designed specifically to improve robustness to background variability in cases where training spectra of the background, as well as the mean spectrum of the analyte, are available. We demonstrate the novel algorithm’s superior performance by comparing quantitative error metrics generated by each method. The experiments consider both simulated data and experimental data acquired from *in vitro* solutions of Raman-enhanced gold-silica nanoparticles.

## Introduction

### Background

Raman spectroscopy is a powerful technique for analyzing chemical compounds using a laser source. It exploits the Raman effect, which arises from the interaction between laser light and a sample of interest. When incoming photons hit the sample surface, most photons are scattered elastically, after which they continue traveling with the same energy and wavelength. However, a very small fraction of the photons is scattered inelastically, meaning that they lose energy and continue traveling with a longer wavelength. The amount of energy lost by the photons depends on the particular molecules they interact with. In fact, the chemical bonds in the molecules absorb energy in highly specific patterns. As a result, the Raman scattered photons possess highly compound-specific wavelength spectra. Raman spectroscopy uses these highly specific spectral fingerprints to identify and quantify compound concentrations. The usefulness and power of Raman spectroscopy lie in the fact that it allows rapid sample analysis of single or multiple compounds (known as multiplexed analysis) at high detection sensitivities [1]. Amongst its many promising areas of application, Raman spectroscopy has gained growing interest from the biomedical research community, where it promises to enable sensitive imaging of nanoparticles for both diagnostic and therapeutic applications [1]–[3]. Examples of such applications are Raman colonoscopy for early cancer detection and improved tumor margin detection during surgery.

A critical part of Raman spectroscopy is the use of an appropriate signal analysis algorithm to analyze the measured Raman spectra. This paper focuses on the development of a signal analysis algorithm that is robust to natural variations in both the background and analyte signal. In the following sections, we describe the previous literature on spectral analysis algorithms, highlight their strengths and weaknesses, and explain the need for and novelty of our contribution.

### Previous Raman Spectral Analysis Algorithms

Various methods have been used to analyze Raman spectra (i.e. detect and quantify compounds of interest), such as classical least squares [1], least squares with a low-order polynomial background model [4], variable baseline correction [5]–[7], explicit detection and parametric (Gaussian) modeling of Raman peaks [8], principal component regression [9], partial least squares [10], [11], and hybrid linear analysis [12]. The classical least squares method can be used when the pure spectra of the compounds of interest (also known as analytes) and an accurate background spectrum are known. While fast and quantitative, this method is sensitive to inaccuracies or variations in the spectra of the compounds of interest and the background. It is also sensitive to noise when the signal of interest is weak. Lutz et. al [4] addressed the problem of background variability by allowing the background spectrum to vary according to a low-order polynomial model. While successful in accounting for slowly varying changes to the background spectrum, it cannot accommodate higher-order variations such as slight peak shifts or changes in the relative amplitudes of peaks, all of which are regularly observed in practice. Many alternative baseline fitting algorithms suffer from the same limitation [5]–[7]. Such methods commonly subtract a smoothly varying baseline estimate from the measured spectra before computing concentration estimates, often using a conventional least squares approach. Again, while suitable to account for smooth background variations such as those caused by autofluorescence, these models are not adequate in the presence of complex sources of variation such as changes in peak position and relative peak amplitudes. Aiming to model such shift and amplitude changes directly, Kode et al. [8] proposed to model spectrum peaks explicitly with 1D Gaussians. By employing a penalized cost function, the method allows the peaks to modestly change position, width and amplitude while computing concentration estimates. However, such peak detection and quantification is sensitive to noise at low signal strengths.

Principal component regression (PCR) and partial least squares (PLS) are implicit methods that require neither prior knowledge of the reference spectra, nor an explicit background model. As such, they can handle background variations that are more complex than the smoothly varying curves discussed above. Known as multivariate calibration methods, they attempt to find a linear model that relates a dependent variable, e.g. analyte concentration, to the measured independent variables, e.g. spectra, for complex mixtures. The model parameters are obtained in the form of a regression or calibration vector *b*, which is derived from a calibration set of representative mixtures for which the dependent variable is known. Subsequently, the dependent variable (analyte concentration) is predicted by taking the dot product of *b* with the measured spectrum of an unknown mixture.

While PCR and PLS are broadly applicable, they also ignore valuable knowledge of the analyte spectra when available. This scenario was encountered previously by Berger et al. [12], who possessed a mixture calibration set with variable spectra, knowledge of the analyte concentration, as well as the pure analyte spectrum. In a bid to improve on PCR and PLS, Berger et al. proposed to exploit the additional information using a method they called hybrid linear analysis (HLA). This technique represents the variable background signal as a linear combination of the background signals’ principal components. It obtains an accurate calibration vector by estimating the background signals (i.e. the calibration signals without the analyte contribution), and subtracting from the known analyte spectrum its projections onto each of the background signals’ principal components (see the section entitled ‘Hybrid Linear Analysis (HLA) Method’). This technique can be repeated to derive the calibration vector for any known analyte in the mixture. HLA was shown to significantly outperform PLS (PCR was not tested because its performance is usually similar but slightly inferior to that of PLS). This result makes intuitive sense because HLA uses more physical information to obtain the calibration vector than PLS (or PCR).

In the study reported here, we possess a calibration set of variable background signals excluding the analyte, as well as a calibration set of pure analyte spectra. The analyte consists of Raman-enhanced gold-silica nanoparticles (see the Simulation Results section). Since the background and analyte variations cannot be modeled by smooth curves (see the Results and Discussion section), multivariate calibration techniques are better suited than baseline correction methods. Of these techniques, HLA is more suitable than PCR or PLS due to the availability of pure analyte spectrum information. HLA can be used by skipping the first step of background isolation (since we measure it directly), and working with the mean of the analyte spectrum. However, such an approach ignores the information about the analyte spectrum variation. It also fails to incorporate information about the *extent* of variation observed in the calibration sets, as captured by the eigenvalues of the principal component analyses.

### Hybrid Least Squares and Principal Component Analysis Algorithm

In this study, we propose to model variations in both the background and analyte spectra, in order to increase the robustness of analyte concentration estimates. Improving on HLA, our method incorporates the principal components as well as eigenvalues of the background and analyte calibration sets into a hybrid least squares and principal component analysis (HLP) method. Our method differs from standard multivariate calibration techniques in that it does not derive a calibration vector. Instead, HLP estimates fitting weights for each of the analyte and the background signals. We explain the mathematical details of the method in the section entitled ‘Novel Hybrid Algorithm (HLP)’. HLP is tested on both simulated data and experimental data acquired from an *in vitro* solution of Raman-enhanced gold-silica nanoparticles [1]. The results are presented in the Results and Discussion section, where we demonstrate the improved performance of the novel method compared to that of the least squares method with a low-order polynomial background model [4] and HLA. To reiterate, we chose HLA as a competitive method for comparison, because it is robust to complex variations in the background signal, and incorporates available information about the analyte spectrum. The latter property makes it more suitable for comparison to our HLP algorithm than PCR or PLS. Lastly, the Conclusions and Further Work section draws conclusions and describes avenues for future work.

## Methods

### Classical Least Squares Method

Here we briefly review the classical least squares method with an added low-order polynomial background model, as proposed by Lutz. et al. [4]. The measured spectrum can be modeled as a linear combination of known spectra (a.k.a. reference spectra):
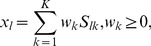
(1)where *x_l_* is the modeled intensity at wavelength *l*, *K* is the number of reference spectra provided, *S_lk_* is the value of the reference spectrum of the *k^th^* compound at wavelength *l*, and *w_k_* is the weight for the *k^th^* compound. In the method proposed by Lutz et al. [4], the spectra *S_k_* include the compounds of interest as well as an average background signal and the *q*+1 components of a *q^th^* order polynomial. The concentrations of the various compounds are then estimated by solving for the weights *w_k_* that give the closest fit with the measured spectrum. This can be done by writing the problem in the matrix form *M* = *SW*, where *M* is the *L*×1 vector containing the measured spectrum values *m_l_*, *S* is the *L*×*K* matrix of reference spectra, and *W* is the *K*×1 matrix containing the weights *w_k_*. The least squares solution is then given by
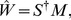
(2)where 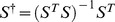 is the Moore-Penrose pseudoinverse of the matrix S. Note that this algorithm implicitly assumes that the noise on the signals is Gaussian distributed.

### Hybrid Linear Analysis (HLA) Method

As mentioned in the section on previous Raman spectral analysis algorithms, the HLA method proposed by Berger et al. [12] estimates the concentration of an analyte by taking the dot product between a calibration vector *b* and a measured spectrum *m*. The calibration vector *b* is computed from a calibration set of mixture spectra, for each of which the analyte concentration is known, as well as an accurate estimate of the pure analyte spectrum. Let *B_A_* denote the calibration set, where the sample spectra are stored in the rows. The corresponding concentrations are stored in a column vector *k*. The known spectrum of the analyte is represented by a row vector *S_A_*, measured at unit concentration. Using *B_A_*, *k* and *S_A_*, the calibration vector *b* is computed as follows:

1. Isolate the background signals by subtracting out the estimated spectral contributions of the analyte:

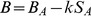
(3)
2. Compute the principal components of the background calibration set *B*, and store them in the rows of a matrix *V*. Since all of the spectra in *B* can be modeled by those in *V*, the spectra in *V* act like pure spectra of the background species.
3. Subtract from the reference spectrum *S_A_* its projections onto each of the background’s principal components, leaving a residual spectrum *r*:

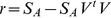
(4)
  This residual *r* is the portion of *S_A_* that cannot be modeled by the spectra contained in *V*, i.e. it is orthogonal to *V*.
4. Normalize *r* to get the calibration vector *b*:

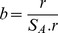
(5)where ‘.’ denotes the dot product.

This procedure can be repeated for any analyte of interest. As mentioned in the section on previous Raman spectral analysis algorithms, HLA can be applied to our data by skipping step (i), and using the mean spectrum of the analyte calibration set.

### Novel Hybrid Algorithm (HLP)

Here we explain our proposed HLP method. It is derived by extending the signal model of Eqn. 1. More specifically, we allow each of the reference spectra *S_k_* to vary according to the principal components of variation observed in the background and analyte calibration sets. For each reference spectrum, we penalize deviations from the mean signal in accordance with the eigenvalues obtained from the principal component analyses. In essence, this constrains the variations in the reference spectra to the statistically plausible. Note that the reference spectra can also include the terms of a low-order polynomial background model. However, this is unnecessary since spectrum variations are already modeled by the principal components. In exploratory experiments (not reported here), we verified that the inclusion of a polynomial background model into HLP did not yield further improvements in concentration estimates.

Mathematically, the signal model is extended to
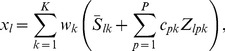
(6)where 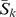 is the mean spectrum observed for compound *k*, and *Z_pk_* is the *p^th^* principal component with a non-zero eigenvalue for compound *k*, observed during prior characterization experiments. Our objective is now to estimate the coefficients 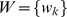 and 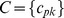 that give the best signal fit. We do so by using a Bayesian probability framework, where we maximize the posterior probability of *W* and *C*, given the measured signal *M*, the mean reference spectra 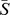, the principal components *Z*, and the eigenvalues 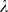 (obtained during the same principal component analysis). Using Bayes’ theorem and the rules of conditional probability, we can decompose this posterior probability as
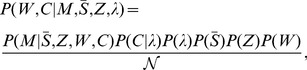
(7)where 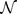 is a normalization constant, and we recognized that 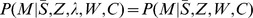. We also assumed that 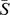, Z, W, and C are statistically independent of each other. Note, however, that C and 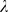 are not assumed to be statistically independent (see Eqn. 10).

The first term of Eqn. 7 is the data likelihood term. Assuming statistical independence of the samples at each wavelength, it is given by
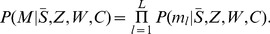
(8)Assuming a zero-mean Gaussian noise model, the probabilities 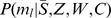 are given by
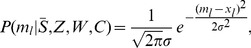
(9)where 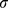 is the standard deviation of the noise in the measured signal. It can be estimated by taking repeated measurements of the same location on a given sample. For the second term in Eqn. 7, we assume that the coefficients 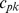 are independent, which yields
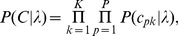
(10)where P is the number of non-zero eigenvalues. In practice, only the first few eigenvalues and principal components are needed, since they already capture most of the variation seen in the calibration sets. In our experiments, the final concentration estimates were found not to be sensitive to the particular number of principal components used as soon as this number exceeded three or four. By definition, the eigenvalues 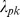 obtained by the principal component analysis are equal to the variance of the coordinates obtained when projecting all data points on the principal component axis corresponding to 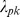, i.e. 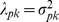. Hence we have
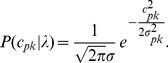
(11)Next, the prior probability functions 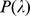, 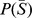, and 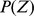 do not contain the variables W and C, and therefore play no part in the optimization problem. Lastly, we assume a uniform distribution for 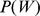.

Following standard practice in optimization problems, we optimize the logarithm of the Bayesian cost function given in Eqn. 7. This simplifies the optimization problem by converting multiplications into summations. After dropping constant terms and cancelling common factors, the cost function can be reduced to
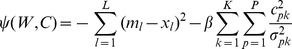
(12)where 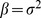. Note that Eqn. 12 takes the familiar form of a penalized maximum likelihood (PML) problem, where 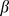 functions as the hyperparameter.

The cost function in Eqn. 12 can be efficiently optimized (maximized) by alternatingly solving it as a standard least squares problem in *W*, and a Tikhonov regularized least squares problem in *C*. Convergence was observed in all experiments after on the order of 100 iterations. For the experiments in the Results and Discussion section, where 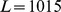, the time per iteration was 0.02 seconds. All coefficients were initialized with a zero initial guess. The expression for the update of the coefficients 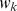 is, similar to the one in the ‘Classical Least Squares Method’ section, given by
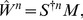
(13)where 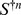 is the Moore-Penrose pseudoinverse of the *n^th^* estimate of the 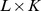 matrix *S*, which is composed of the signals 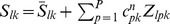, where 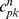 are the latest estimates of 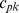. To obtain the update steps for the coefficients 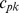, it is instructive to substitute Eqn. 6 into Eqn. 12, and to rewrite the latter as
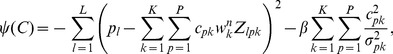
(14)where 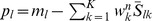, and 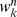 are the latest estimates of 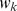. To formulate our update step, we store the elements 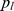 into an 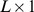 vector Q, and the elements 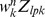 in an 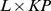 matrix 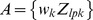. The matrix C is of size 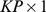. Maximizing Eqn. 14 is then equivalent to minimizing the cost function
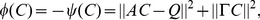
(15)where 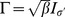, and 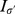 is a 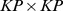 diagonal matrix that contains the values 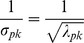 along its diagonal entries. Eqn. 15 is a standard Tikhonov regularized least squares problem, and has the explicit solution



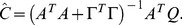
(16)To complete this dicussion, we would like to highlight once again the two key differences between HLA and HLP. First, HLA does not account for variability in the analyte spectrum, whereas HLP does. Second, HLA does not take into account the eigenvalues of the principal component analyses, while they represent important information about the magnitude of the variations observed in the calibration sets. HLP improves on HLA by using the eigenvalues within a Bayesian statistical framework to regularize the weights 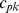, and thereby to constrain the allowed variations about the mean spectra to a well-justified statistical range. The resulting improved performance of HLP over HLA is demonstrated in the Results and Discussion section.

## Results and Discussion

Here we compare the performance of HLP to that of least squares with a third-order polynomial background model (from hereon referred to as LS-3P), as well as HLA. The third order polynomial was found to give optimal results for Raman signals similar to the ones used here by Lutz et al. [4]. In this paper, we restrict our attention to the case where *K* = 2. Higher values of *K* are deferred to future work (see the Further Work section). In other words, the measured spectra are modeled by two reference spectra - one for the analyte (in our case gold-silica nanoparticles), and one for the background. We compare the performance of LS-3P, HLA and HLP for various relative strengths of the analyte and background signal.

### Simulation Results

**Figure 1:**
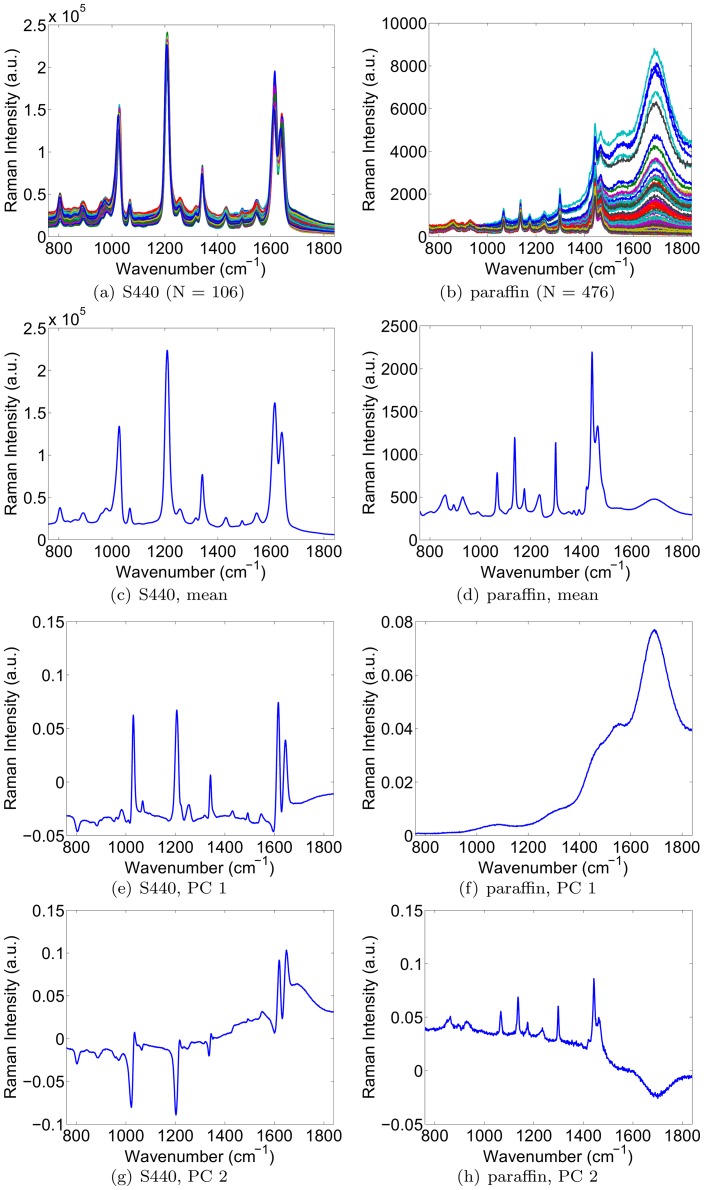
Acquired signals, mean signal, and the first two principal components of the S440 nanoparticle (left) and the paraffin background (right).

In this section, we simulated the presence of a signal of interest (analyte signal) within a background signal. Our aim was to characterize the accuracy with which the weight (or concentration) of the signal of interest could be recovered, in spite of variability in both the signal of interest and background signal. This analysis was repeated for various dynamic ratios of the two signals. To obtain source signals with a realistic degree of variability to use in the simulation, we collected Raman spectroscopy signals from a real 0.8 nM solution of Raman-enhanced S440 gold-silica nanoparticles produced by Oxonica (now owned by Cabot Security Systems, Boston, MA, USA), as well as signals from a paraffin background material. By performing raster scans across the solution as well as background material, we obtained 106 signals for the S440 nanoparticle solution, and 476 signals for the paraffin background. The collection of signals, the mean signal, and the first two principal components for each are shown in Fig. 1. Note that the bump in the mean paraffin spectrum in the 1600–1800 range appears smaller than could be expected from Fig. 1(b). This is simply due to the fact that the majority of the 476 paraffin spectra contain a smaller bump than the 30 or 40 outlying spectra that visually dominate the plot in Fig. 1(b).

The characterization study was performed by conducting a sequence of experiments as follows. In each experiment, we picked one S440 signal and one paraffin background signal from our database. We then simulated a measured signal by weighting the chosen S440 signal and adding it to the chosen paraffin background signal. This was done for S440 weights of 2^0^, 2^−1^, …, 2^−12^, and 2^−13^. The background signals were not weighted. This was done for all possible combinations of S440 and paraffin signals, at each concentration of S440, yielding a total number of 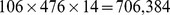 simulated signals. For each combination of an S440 and paraffin signal, the remaining signals in the database were used to compute the mean S440 and background signals, as well as their respective principal components. The signal strength (weight 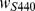) of S440 was then recovered by the LS-3P, HLA and HLP algorithms. Finally, the recovered weight 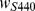 was converted to a concentration estimate 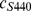 using the formula 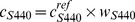, where 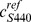 was the concentration of the S440 solution from which the reference spectrum was measured (in this case 0.8 nM).

The performance of each method was evaluated using two metrics: one to measure the closeness of the fit, and one to measure the accuracy of the concentration estimates themselves. The former was the Durbin-Watson statistic of the residual error, as defined by
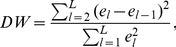
(17)where 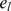 is the residual error at the 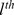 wavenumber. The Durbin-Watson statistic can take values from 0 to 4. A value of 2 signifies no autocorrelation between the successive error values. Values substantially less than 2 indicate positive serial correlation, while those substantially larger than 2 indicate negative serial correlation between the error values. The latter metric was the fractional error, defined as 
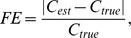
 where 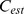 and 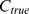 are the estimated and true concentrations of the S440 signal.

**Figure 2:**
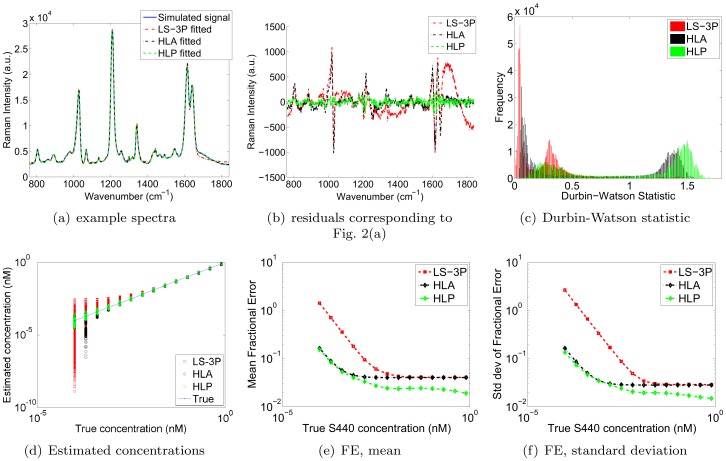
Simulation results. (a) Example of fitted spectra, (b) corresponding residuals, (c) histograms of Durbin-Watson statistics produced by each method, (d) estimated concentrations, and (e-f) mean and standard deviation of fractional errors generated by the LS-3P, HLA and HLP algorithms.

An example spectrum, as well as the fitted spectra by LS-3P, HLA and HLP are shown in Fig. 2(a). Note that the fitted LS-3P spectrum shows a wiggle in the 1650–1800 wavenumber range. This is caused by over-fitting of the 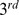 order polynomial background model, which tries to compensate for the variable magnitude of the bump in the 1600–1800 wavenumber range of the paraffin spectrum. As such, the fitted LS-3P spectrum illustrates the limited ability of polynomials to model irregular background variations. While higher order polynomials are able to capture sharper and more irregular background variations, they suffer from increasing degrees of over-fitting and “whiplash” effects near the sides of fitted spectra. Lower order polynomials are better behaved, but can account only for slow and smooth background variations. The 3*^rd^* order polynomial model was shown to offer a good compromise between these effects for similar data in [4], but the above mentioned wiggle still reveals its limitations. In essence, the plot illustrates that the variations present in the S440 and paraffin spectra are poorly modeled by a standard polynomial model. From a visual inspection of Fig. 2(a), both HLA and HLP handle the variability more effectively and provide better fits than LS-3P.

Fig. 2(b) shows the residual errors of the fits shown in Fig. 2(a). LS-3P clearly yields the greatest residual errors, indicating a failure to model the variations in the component spectra. HLA captures more of the background variation, but not as much as HLP, which shows the smallest residual errors. Fig. 2(c) confirms this trend across all fitted spectra, with superimposed histograms of the Durbin-Watson statistics produced by LS-3P (red), HLA (black), and HLP (green). The histograms show that all three algorithms display positive serial correlation (DW < 2). However, the positive serial correlation of LS-3P tends to be highest (DW values closest to zero), and that of HLP tends to be lowest (DW values closest to two). HLA yields slightly greater degrees of positive serial correlation than HLP, but much less so than LS-3P. Note also that the shape of the DW histograms was similar for all three methods, with each distribution exhibiting two peaks. We found that a particular analyzed spectrum occupied the same relative position in the histograms for each method. In other words, the shifts in the histograms shown in Fig. 2(b) reflect shifts that were true for all of the individual analysed spectra. In short, HLP provided the most right-shifted histogram, and hence yielded the lowest positive serial correlation for all spectra.

Next, Fig. 2(d) shows the estimated concentrations by LS-3P, HLA and HLP, revealing that HLP outperforms both LS-3P and HLA, in the sense that the spread around the true curve is least for HLP. One can also see that HLA produced a large downwards deviation from the true concentration for the second lowest true concentration. This apparent instability yielded 65 values that were lower than the lowest LS-3P value for that concentration. While these 65 cases represent only a small fraction of the total number of signals analyzed, it is worth noting that HLP did not suffer from such instability for *any* of the analyzed spectra. Next, the curves in Fig. 2(e-f) show the means and standard deviations of the fractional errors as a function of true S440 concentration for both algorithms. The above mentioned instability did not significantly affect HLA’s performance curves because the number of signals involved was relatively small. Once again, the HLP algorithm clearly outperforms the LS-3P algorithm at all S440 concentrations, and most markedly so at lower S440 signals/concentrations. It also outperforms HLA across the range of concentrations tried, doing so most markedly at higher concentrations. Presumably this is because HLP models the variations in the analyte spectrum, whereas HLA does not.

### Experimental Results

#### Variable background phantom

**Figure 3:**
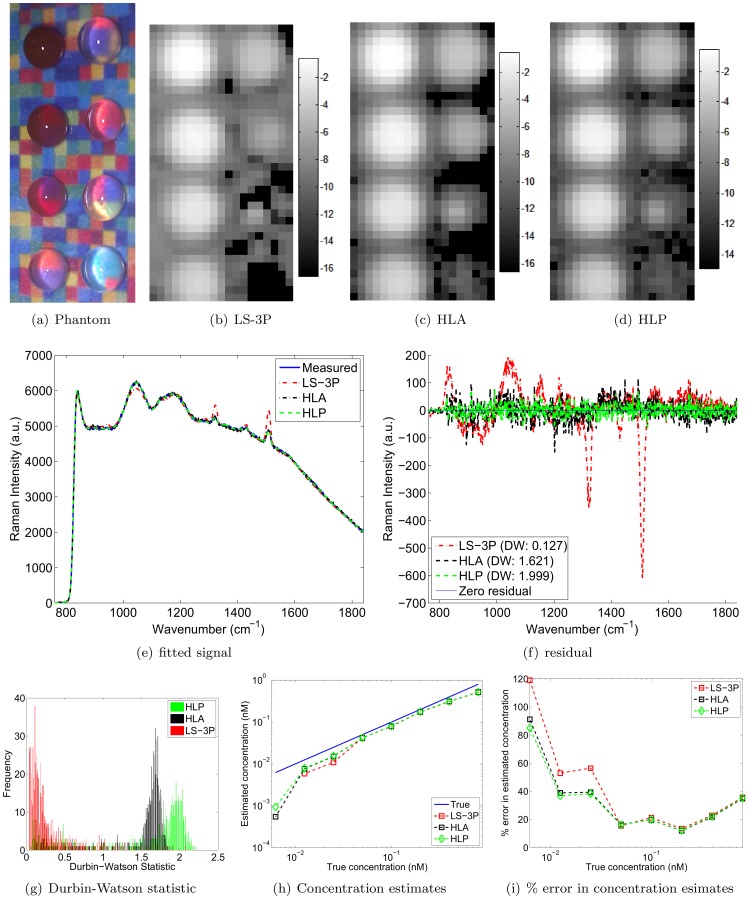
Color background results. (a) Phantom, (b-d) log2 of S440 concentrations by LS-3P, HLA, and HLP, (e-f) example fitted spectra and residuals by LS-3P, HLA and HLP, (g) histogram of the Durbin-Watson statistic for all pixels, and (h-i) quantification of concentration estimation accuracy. ‘True’ in (h) plots the theoretical linear relationship between the estimated and true concentrations of S440. The concentration estimate by LS-3P for the lowest true concentration was negative and hence not shown in the logarithmic plot in (h).

**Figure 4:**
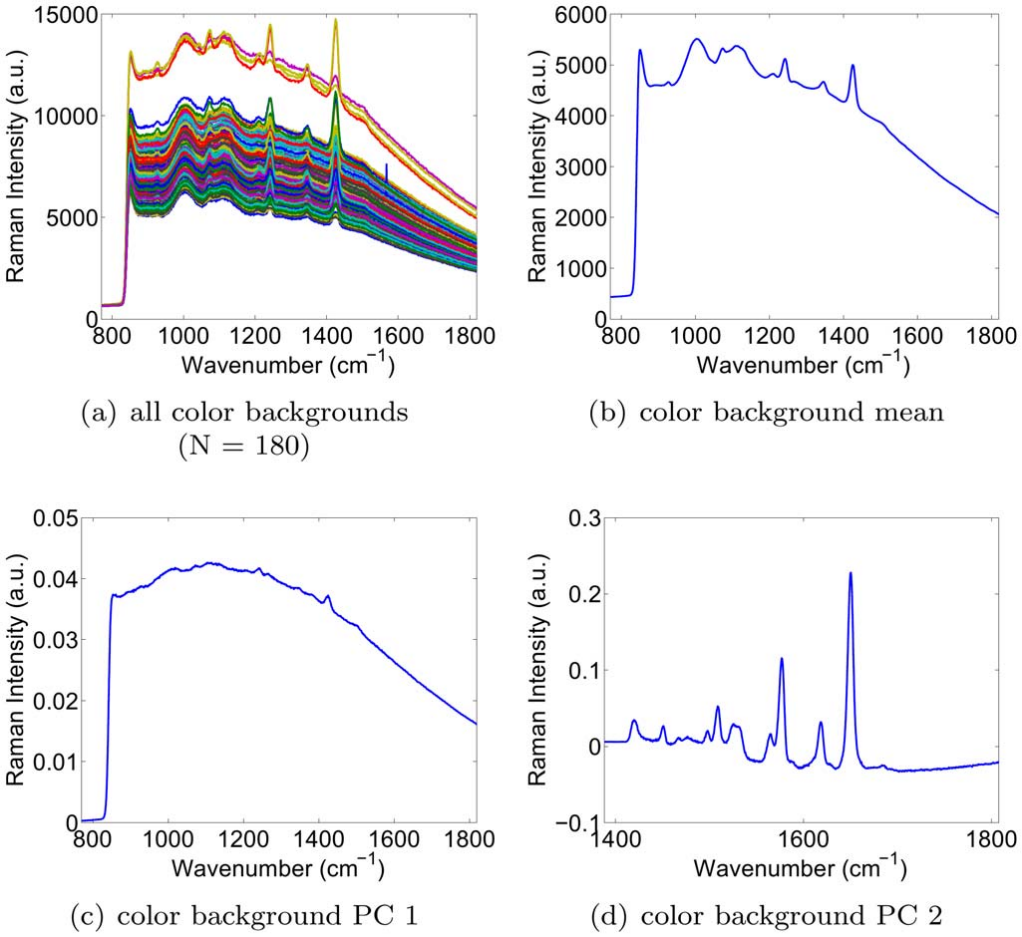
Background signals of the printed colors: mean and first two principal components.

To demonstrate that HLP outperforms LS-3P and HLA on experimental data as well, we designed an experimental phantom where the background signal varied significantly. We placed eight drops of decreasing concentrations of S440 nanoparticles on a thin film of paraffin, which was in turn placed on a background of various colors (see Fig. 3(a)). This background was obtained by printing a color image of a matrix of random numbers between 0 and 1. These printed colors each possessed a distinct Raman spectrum. The first S440 drop had a concentration of 0.8 nM, and subsequent drops were obtained by each time halving the concentration. Raman spectra were acquired on a Renishaw InVia Raman microscope, which was modified by our laboratory for biomedical applications [13]. The integration time of each acquisition was 1 second, and the laser had a wavelength and power of 785 nm and 15 mW, respectively. To characterize the background signal, we first performed a raster scan of the printed color background. The background was covered with a thin paraffin film and had several blank drops of suspension solution (distilled water) placed on top of it. The collection of all acquired signals, as well as the mean background signal and the first two principal components are shown in Fig. 4. The logarithm-transformed (base 2) images of the estimated S440 concentrations are shown in Fig. 3(b-d) for the LS-3P, HLA and HLP algorithms, respectively. The black pixels in the images are points for which the algorithms produced negative weights. It is clear that HLA and HLP succeed in imaging the lowest concentration drop (bottom right), whereas LS-3P breaks down at this low concentration.

To further examine the difference between the three methods, and between HLA and HLP in particular, we provided fitted spectra as well as quantitative results in Figs. 3(e)-(i). Fig. 3(e) shows example fitted spectra computed by LS-3P, HLA and HLP. Fig. 3(f) shows the residuals, obtained by subtracting the fitted spectrum from the measured spectrum. In this example, HLP shows hardly any serial correlation in the residual error (Durbin-Watson = 1.999), unlike LS-3P, which shows clearly structured residuals (Durbin-Watson = 0.127). It also compares favorably to the Durbin-Watson statistic of 1.621 by HLA. Fig. 3(g) shows that the favorable Durbin-Watson statistic of HLP over LS-3P and HLA generally holds for all spectra. HLP achieves values closest to 2, which indicates zero serial correlation. HLA again gives the second best performance. In other words, HLP best captures the variability in the background signals, translating into the closest fits, and the least amount of serial correlation in the residual errors. Note also that the distribution of the Durbin-Watson statistic takes a different shape from the one seen in Fig. 2(c); Fig. 3(c) shows only one peak, whereas Fig. 2(c) shows two peaks. The difference is merely due to the presence of different background materials. The conclusion is however unchanged: HLP right-shifts the entire distribution towards a DW value of 2.

An evaluation of the accuracy of each method in terms of their concentration estimates is provided by Figs. 3(h) and (i). The former shows the estimated concentrations of the droplets; the latter shows the percent error in the estimated concentrations. The concentration estimates were computed by taking the average of the 6 brightest pixels within each droplet. The curves show that LS-3P, HLA and HLP perform very similarly for high nanoparticle concentrations. The performance of LS-3P deteriorates the most rapidly of the three algorithms as the nanoparticle concentration decreases. HLA and HLP yield similar results, though HLP yields better estimates towards the lower nanoparticle concentrations, i.e. when the nanoparticle signal strength is low relative to the background signal. Note that the lowest weight was not shown for LS-3P since it was negative, and hence does not have a real-valued logarithm.

#### *Ex vivo* pig colon experiments

**Figure 5:**
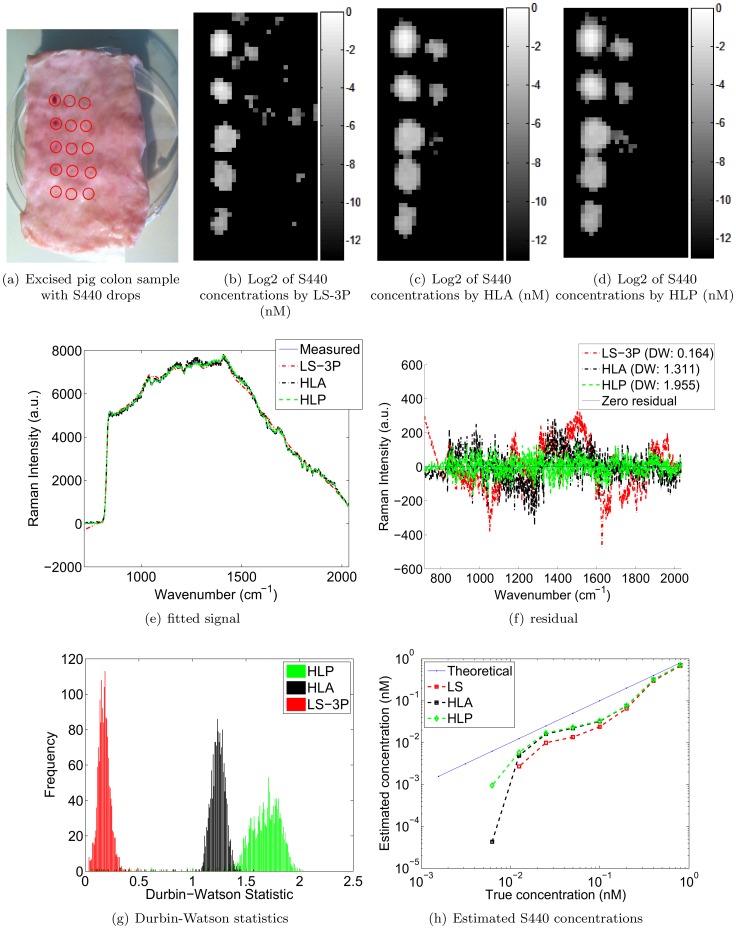
Excised pig colon results.

**Figure 6:**
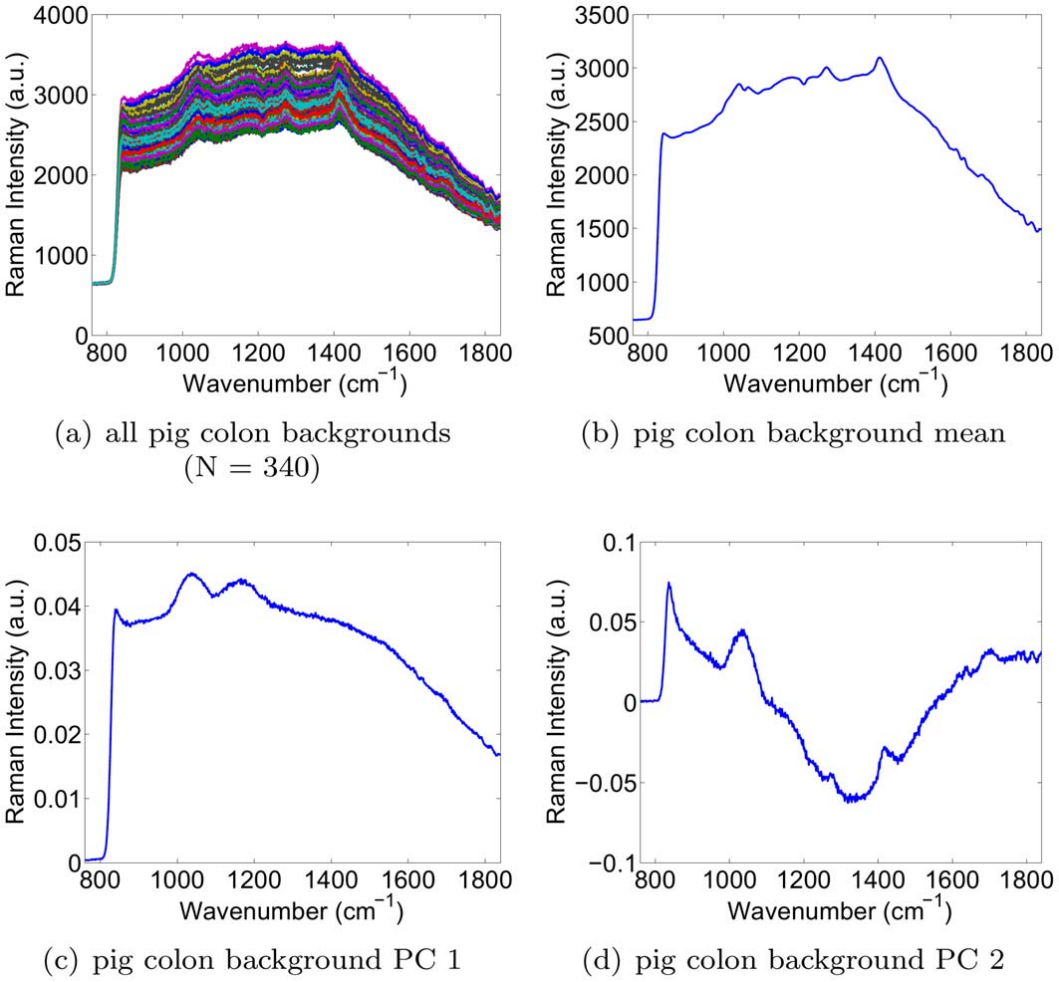
Excised pig colon backgrounds, mean, and first two principal components.

In this experiment, we placed S440 drops of decreasing concentrations onto an excised pig colon as shown in Fig. 5(a). All drop locations are identified by a red circle. The pink dots within the red circles reveal the locations of the most concentrated S440 drops. The drops were produced by starting with a stock concentration of 0.8 nM, and serially diluting by a factor of 2 for every subsequent drop. Drops were arranged in 3 columns of 5 drops, starting on the top left of the sample. The drop concentrations decreased down the columns. Next, we raster scanned the sample with a 1 mm step size, a 1 second integration time, and a 785 nm laser with a power of 15 mW. The total time for this scan was approximately 1 hour. Prior to the placement of the drops, the pig colon was also raster scanned at a step size of 2 mm to obtain a library of background signals. The obtained background signals, as well as their mean and first two principal components are shown in Fig. 6. The objective of the experiment was to compare the S440 concentrations computed by LS-3P, HLA and HLP. The calibration spectra for S440 were obtained at the stock concentration of 0.8 nM. Figs. 5(b-d) show the concentration maps obtained by LS-3P, HLA, and HLP, respectively. HLP and HLA both eliminated the false positive weights generated by LS-3P in the tissue background where no S440 is present. However, HLP captured more of the lowest concentration drop than HLA. Figs. 5(e-f) show example fitted spectra with their corresponding error residuals. It is clear that HLP produced the closest fits, as evidenced by the lowest amount of structure in the error residuals. Fig. 5(g) shows a histogram of the Durbin-Watson statistics for all fits by each algorithm. The HLP histogram shows values that are closest to 2, confirming that HLP’s closer fits held widely across all measured spectra.

Lastly, Fig. 5(h) plots the S440 concentrations computed by each algorithm. The concentrations were computed by taking the mean concentration of the 5 brightest pixels in each drop. Note that the S440 particles were not detectable beyond the seventh drop for LS-3P, and not beyond the 8th drop for HLA and HLP. The concentration estimates computed by HLP adhered most closely to the expected linear relationship (as shown by the blue ‘Theoretical’ line). As before, HLA gave the second best performance, and LS-3P gave the poorest performance.

### Conclusions and Further Work

#### Conclusions

In this work, we showed that the LS-3P algorithm for Raman spectroscopy is sensitive to natural variations in the reference spectra. Our compound of interest was a solution of Raman-enhanced gold-silica nanoparticles. We found that the nanoparticle concentration estimates were sensitive to spectrum variability primarily when the nanoparticle signal (concentration) was weak. We proposed a novel algorithm (HLP) that is more robust to variations in the reference spectra. The HLP method was compared to both LS-3P and Berger et al.’s HLA method [12], where the latter was specifically designed as an improvement over PCR and PLS, and to be robust to variability in the background signal. HLP’s superior performance over LS-3P and HLA was shown for both simulated and experimental data. The simulated data was generated by digitally combining weighted instances of experimentally obtained nanoparticle and background spectra. The experimental data was obtained from serially diluted *in vitro* solutions of Raman-enhanced gold-silica nanoparticles. We confirmed our expectation that Berger et al.’s HLA method significantly outperforms LS-3P. However, our HLP method improved concentration estimates even further for two reasons. First, unlike HLA, it accounts for variability in the reference spectrum of the nanoparticle *itself*, not just in background signals. This is a useful property since, in practice, a single reference spectrum will always contain inaccuracies. Second, HLP incorporates the eigenvalues of the principal component analysis. This regularizes how far the reference spectra are allowed to ‘stray’ from their means, in accordance with experimentally observed variability. Both of these modeling improvements result in the improved performance of HLP compared to HLA and LS-3P, as demonstrated by the experiments in this paper. We also note that our performance metrics (Durbin-Watson statistic and fractional error) were computed over many spectrum instances (N = 14×106×476 = 706,384 for the simulated data, N = 180 for the color background experiment, and N = 1,653 for the pig background experiment). In other words, our conclusion that HLP outperforms HLA is strengthened by the fact that the improvement was observed across many different spectra. Furthermore, the improvement was demonstrated across three different experimental set-ups, lending further credibility to our results. Lastly, the improvement makes intuitive sense because of the additional information exploited by HLP compared to HLA, namely the eigenvalues of the background calibration set, and the principal components and eigenvalues of the analyte calibration set.

#### Further work

In this paper, the HLP algorithm was evaluated for the case where *K* = 2, i.e. where only a single analyte and mean background were considered. In future work, we will examine the performance of HLP for multiplexed spectroscopy (*K*>2). Second, the HLP algorithm presented in this paper assumed a Gaussian noise distribution. In our future work, we will evaluate the merits of using a Poisson instead of Gaussian noise model, in an effort to decrease the lowest detectable nanoparticle concentration. Lastly, we recall that the HLP algorithm is equally capable of modeling variations in the background spectrum as in the spectra of compounds of interest such as gold-silica nanoparticles. While the S440 gold-silica nanoparticle spectra were found to be relatively stable in this study, other studies have reported significant variability in the spectra of nanoparticle solutions (eg. [14]). A common cause for such variability is non-uniformity in the particle sizes. In future work, we will examine HLP’s ability to improve the robustness of the concentration estimates for such nanoparticles with less stable spectra. Lastly, we aim to investigate the spectral variability of biomolecules as well, due to effects such as conformational changes. As before, we will assess the extent of those variations, as well as the robustness of our novel HLP method to them.
